# Polyaniline/(Ta_2_O_5_–SnO_2_) hybrid nanocomposite for efficient room temperature CO gas sensing†

**DOI:** 10.1039/d2ra00602b

**Published:** 2022-05-25

**Authors:** Chethana Aranthady, Ganapati V. Shanbhag, Nalini G. Sundaram

**Affiliations:** Materials Science and Catalysis Division, Poornaprajna Institute of Scientific Research Devanahalli-562164 Bengaluru India nalini@poornaprajna.org; Graduate Studies, Manipal Academy of Higher Education Manipal-576104 Karnataka India; Department of Chemistry, St. Josephs's College (Autonomous) Bengaluru-560027 India

## Abstract

Development of efficient CO sensors that can detect low concentration CO at room temperature is of prime importance. Herein, we present a Ta_2_O_5_–SnO_2_–PANI hybrid composite for the efficient sensing of CO at room temperature and at very low concentrations. The material was synthesized by the oxidative polymerization method. The structural and morphological characteristics of the nanostructured (Ta_2_O_5_–SnO_2_)–PANI hybrid composite were examined using p-XRD and FESEM techniques. The oxygen vacancies in the material were confirmed by XPS analysis. The hybrid material exhibited superior CO sensing performance with high sensitivity, low operating temperature, and fast response and recovery time compared to the individual counterparts. The enhanced sensing ability of the hybrid material is accredited to the synergistic properties such as conductivity of PANI, improved oxygen vacancies and the heterostructure formed between the PANI and the (Ta_2_O_5_–SnO_2_) composite. These remarkable features make TaSn : PANI a potential sensor at room temperature for sensing of low concentration CO.

## Abstract 2

Room temperature CO sensing using a hybrid (Ta_2_O_5_–SnO_2_) : PANI composite material.

## Graphical abstract

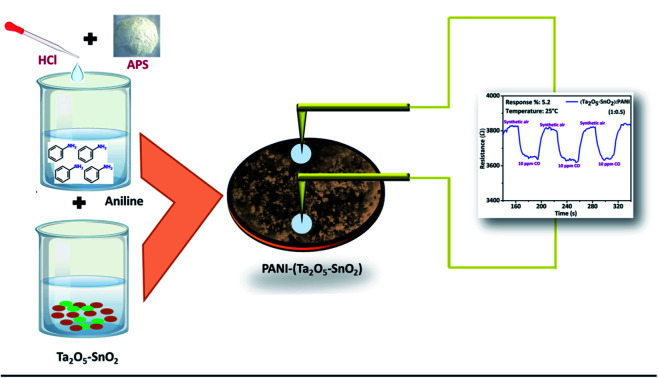

## 1. Introduction

The widespread use of fossil fuels tends to produce hazardous gases like CO, CO_2_, CH_4_, SO_2_, NO_*x*_, and other hydrocarbons which results in severe environmental pollution. It is reported that the toxic gases CO and NO_*x*_ on reacting with sunlight produce O_3_ which is harmful to living beings.^1–4^ Among the various air pollutants, CO is one of the most toxic gases which causes catastrophic effects on mankind. Studies have proven that human blood has more affinity (250–300 times) to CO than oxygen. Further, hemoglobin in the blood forms a complex with CO and causes a decrease in the oxygen-carrying capacity of the blood. The World Health Organization (WHO) has assigned the TLV (threshold limit value) limit of CO as 10 ppm for 8 h exposure,^5^ above which would lead to lethal damage. Therefore, rapid detection of CO gas at a lower concentration at early stages of exposure is of utmost importance. There is intense ongoing and future research for the development of novel sensors that meet the aforementioned characteristic traits and exhibit remarkable sensing performance compared with the existing gas sensing materials.^6–8^

The metal oxide sensors are at the forefront of gas sensing technology owing to their desirable properties such as high surface active sites, high surface to volume ratio and strong adsorption on the surface.^9,10^ Nevertheless, the design of metal oxides based sensors for room temperature gas sensing is still an arduous task. Therefore, exploring new strategy to improve the sensitivity is very important.^11^ Research studies have found that combination of organic–inorganic hybrid nanocomposites would overcome these limitations. This would harness the ensemble effect of organic materials with that of inorganic metal oxides and help in realizing the metal oxide based sensors at room temperature. In this context, conducting polymers have emerged as promising materials that help in lowering operating temperatures of gas sensors.^12,13^ Introducing inorganic metal oxide in a polymer matrix helps to improve the stability, mechanical and conducting properties of the sensor.^14^ They improve sensitivity and selectivity by enabling specific binding sites.^15^ Conducting polymers have inherent porous structure and large surface area as they are made up of planar structures. Further, they act as channels for charge transport.^13,16^ They have exceptional electrical properties due to the delocalization of π-electrons throughout the polymer chain.^17,18^

In recent years, researchers explored variety of conducting polymers forming hybrid composite with metal oxides giving rise to excellent sensing performance. For instance, Qu Hu *et al.* have demonstrated room temperature sensing of ammonia gas (10 ppm) using composite of polyaniline–NiO.^19^ Shubha *et al.* reported a hybrid composite of poly(3,4-ethylenedioxythiophene : poly(styrene sulfonic acid)) (p-PEDOT–PSS) and (n-TiO_2_) to detect NO gas (1–250 ppm) at room temperature.^20^ Further, Yang *et al.* observed room temperature sensing of 5 ppm NO_2_ gas using hybrid composite of poly(3-hexylthiophene) (P_3_HT) and (ZnO-graphene oxide) nanoparticles.^21^ Similarly, Yang Li *et al.* used polypyrrole–ZnO composite to sense NH_3_ gas (0.5 ppm) at room temperature.^22^ Among the various polymers, polyaniline is of particular interest for sensing applications owing to its versatile properties such as tunable electronic properties, good electron conductivity, rich redox properties, ease of polymerization, and high stability at ambient conditions.^11,12,23^

However, as such employing polyaniline alone results in poor mechanical stability and chemical sensitivity, long term stability issues, irreversible response, poor selectivity, long response and recovery time as compared to metal oxides.^24,25^ Therefore, incorporating metal oxides into PANI matrix may lead to new electronic interactions, charge transfers, morphological modifications. A combination of these effects could help in significance improvement in the sensing properties and better gas sensing performance. The hybrid material not only improves the existing properties, it also exhibits new properties characteristic to the hybrid material.^12,26–28^

In this direction, several works have been reported for the detection of various toxic gases.^16,26^ In particular, Sen *et al.* reported room temperature sensing of 75 ppm CO using polyaniline Co_3_O_4_ composite.^29^ Similarly, Atanu Roy *et al.* have studied room temperature CO sensing (500–1000 ppm) of PANI-multiwalled carbon nanotube.^30^ These pioneering works on polyaniline composites have motivated further research to develop novel room temperature sensors to detect low ppm CO gas. Various metal oxides have been used for the detection of CO gas due to their good sensing properties, high stability, *etc.* Very small amount of the metal oxide is sufficient to bring about desired improvement in properties. Hence, this is considered as an efficient and economical method.

In the present work, we synthesized a hybrid organic–inorganic composite using PANI and (Ta_2_O_5_–SnO_2_) (henceforth referred as TaSn : PANI). The hybrid composite of (Ta_2_O_5_–SnO_2_) and PANI was synthesized by oxidative polymerization complex method and characterized by various techniques. Interestingly, it exhibited remarkable sensing performance to detect low concentration CO (up to 1 ppm) at room temperature. The sensor performance was superior to the inorganic composite (Ta_2_O_5_–SnO_2_) in terms of its operating temperature, sensitivity, response and recovery time. The appealing features of this work includes simple material design, good sensitivity and selectivity to low concentration CO, reproducibility, low operating temperature and low power usage. This study opens up a new avenue in designing a novel sensor which operates at ambient condition to detect low concentration CO.

## 2. Experimental

### 2.1 Synthesis of (Ta_2_O_5_–SnO_2_) nanocomposite

Ta_2_O_5_–SnO_2_ composite was synthesized by hydrothermal method. Metal precursors of SnCl_4_·5H_2_O and TaCl_5_ were dissolved in 30 ml distilled water and stirred for 2 h. NH_4_OH was added to this solution and pH was maintained at 9. The reaction mixture was transferred to a Teflon-lined stainless steel autoclave and heated at 200 °C for 6 h. After cooling, it was washed with water and ethanol and dried at 120 °C for 2 h. The material was further calcined at 700 °C to form the composite.^31,65^

### 2.2 Synthesis of hybrid (Ta_2_O_5_–SnO_2_)–PANI composite

A known amount of aniline was added into a beaker containing 30 ml of distilled water. HCl was added to this solution to adjust the concentration of H^+^ to 1 mol L^−1^ by HCl. The solution was sonicated for 30 minutes and 0.5 g of (Ta_2_O_5_–SnO_2_) inorganic composite was added in it. Here, we synthesized 3 batches with different weight ratios of (Ta_2_O_5_–SnO_2_) to aniline (1 : 0.75, 1 : 0.50, 1 : 0.25). After adding the (Ta_2_O_5_–SnO_2_) composite, the mixture was stirred well and sonicated further for about 30 minutes. The resultant mixture was transferred to a Teflon-lined stainless steel autoclave followed by the addition of ammonium peroxydisulfate (APS : aniline molar ratio was 1 : 1). The reaction mixture was heated at 140 °C for 4 h. After cooling, the product was filtered, washed with water and ethanol and dried at 60 °C.^26^ Similarly, PANI alone was synthesized as per the above procedure without adding the inorganic composite.

### 2.3 Material characterizations

Phase purity was confirmed by powder X-ray Diffraction (p-XRD) technique using Bruker D2 Phaser diffractometer with Cu Kα source (wavelength-1.540 Å, 2*θ* range: 10° to 70°, step: 0.02°). Gemini Technology Scanning Electron Microscopy was used to analyze the surface morphology of the hybrid material. The presence of PANI was confirmed by the Fourier Transfer Infrared Spectroscopy using Alpha T-Bruker instrument in the wavenumber range of 4000–500 cm^−1^ by KBr pellet method. Emission spectra of hybrid TaSn : PANI, (Ta_2_O_5_–SnO_2_) and PANI were recorded using Agilent Cary eclipse fluorescence spectrophotometer in the wavelength range 350–600 nm (excitation wavelength: 248 nm). The RAMAN spectroscopic measurements were performed using LabRAM HR RAMAN spectroscopic system in a spectral range 200–2000 cm^−1^ using a visible laser *λ* = 532 nm at room temperature. The chemical compositions and oxygen vacancies were determined by X-ray Photoelectron Spectroscopy (XPS) using AXIS Ultra instrument. For gas sensing measurements, approximately 300 mg of powder samples were pressed into pellets of diameter 13 mm and silver paste was applied on the surface of pellets as electrical contacts. DC probe station with two probes was used for sensing measurements. Gas sensing experiments were performed at an operating voltage of 1 V. Synthetic air was used as carrier gas during the measurements. Alicat Mass Flow Controller (MFC) was used to control the target and carrier gas flow. Change in the resistance of the material during the exposure of the gas was monitored using Keithley source meter-2450. To monitor concentration of the gas and for the dynamic sensing measurements Flowvision and Kickstart softwares were used respectively.

## 3. Results and discussion

### 3.1 Morphology and structural characterization

**Fig. 1:**
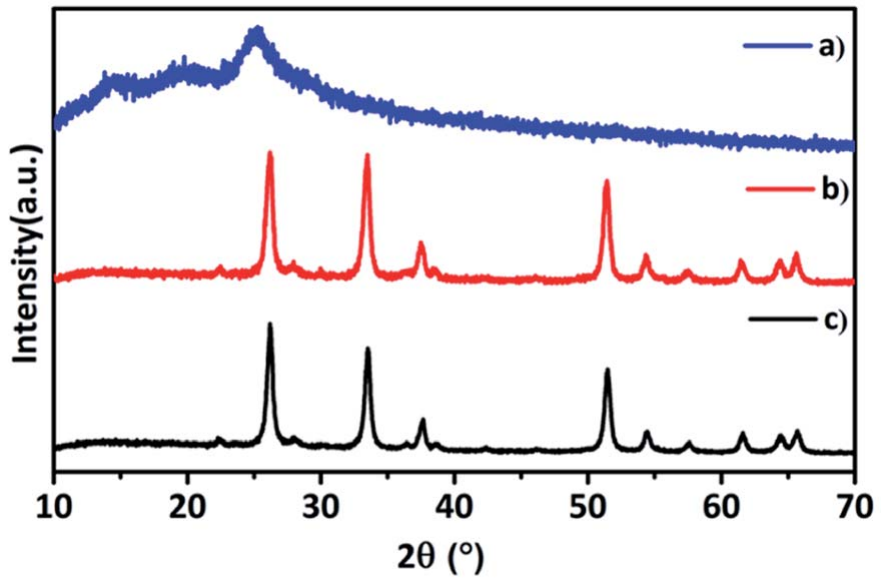
p-XRD patterns of (a) polyaniline (PANI), (b) hybrid TaSn : PANI, (c) (Ta_2_O_5_–SnO_2_) nanocomposite.

From the XRD patterns of hybrid TaSn : PANI and pure PANI and (Ta_2_O_5_–SnO_2_) nanocomposite (Fig. 1), it is observed that the inorganic composite (Ta_2_O_5_–SnO_2_) retained its structure even after mixing with PANI. No impurity peaks or peak shifts were observed in the hybrid material. XRD pattern of hybrid material consists of both SnO_2_ (ICSD no. 191343) and Ta_2_O_5_ (ICSD no. 66366) crystal systems. The amount of polyaniline added was very less and therefore, it was not detected in the p-XRD; however the presence of PANI in the hybrid composite was confirmed by Fourier Transfer Infrared Spectroscopy (FTIR) technique. The powder XRD patterns of other compositions such as TaSn : PANI (1 : 0.25) and TaSn : PANI (1 : 0.75) are given in Fig. S1.†

**Fig. 2:**
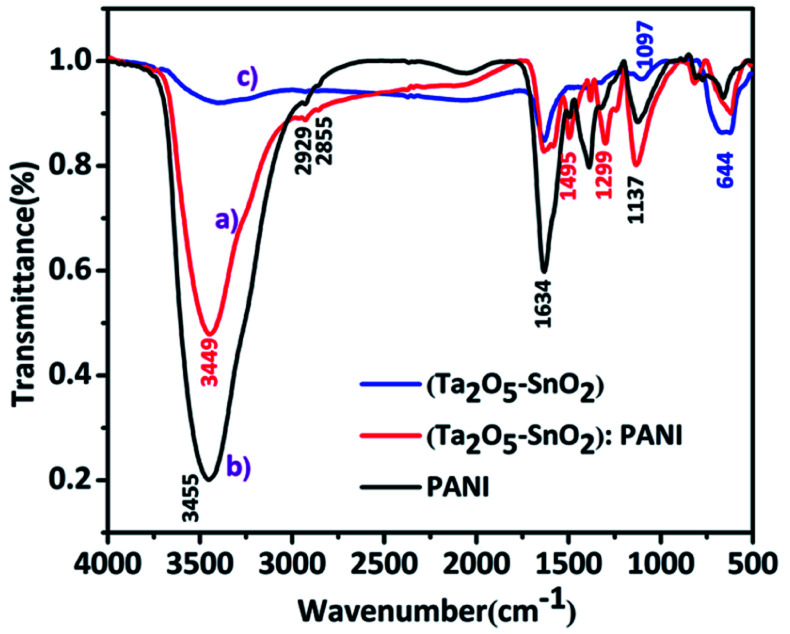
FTIR spectra of (a) TaSn : PANI (1 : 0.50) (b) PANI and (c) (Ta_2_O_5_–SnO_2_)composite.

Fig. 2 shows FTIR spectra of hybrid TaSn : PANI (1 : 0.50) compared with pure (Ta_2_O_5_–SnO_2_) composite and PANI. The peak at 3455–3449 cm^−1^ appears due to the stretching vibrations of N–H bond of polyaniline while the peaks at 1634 cm^−1^ could be attributed to bending vibrations of adsorbed water molecules.^32^ The peak at 1495 cm^−1^ corresponds to the stretching vibrations of C–C bond in benzenoid ring and 1299 cm^−1^ which could be attributed to the stretching vibrations of C–N in benzenoid rings. The strong peak at 1137 cm^−1^ is due to the in-plane bending vibration of benzenoid C–H groups. The broad band at 1097 cm^−1^ is due to the Ta–O vibrations.^33^ A prominent peak at 644 cm^−1^ can be ascribed to the vibrations of Sn–O bond in SnO_2_.^24^

**Fig. 3:**
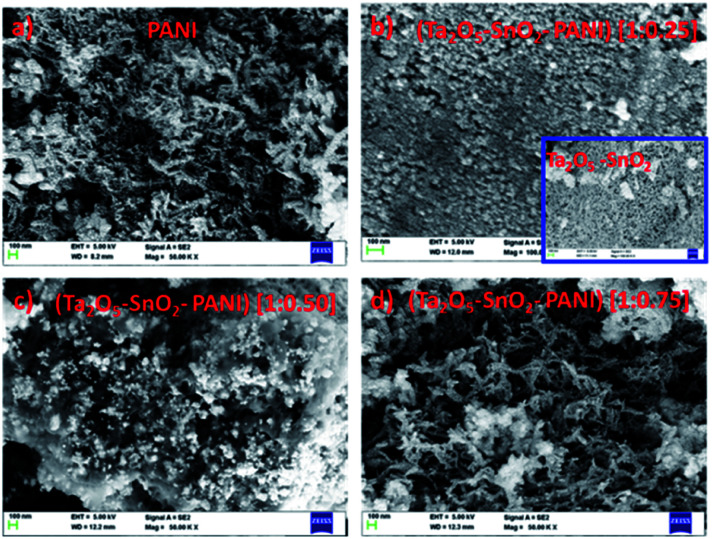
FESEM images of (a) PANI, (b) TaSn : PANI (1 : 0.25), (Ta_2_O_5_–SnO_2_) [inset], (c) TaSn : PANI (1 : 0.50), (d) TaSn : PANI (1 : 0.75).

The FESEM image (Fig. 3a) of PANI consists of cluster of fibrous network like structure. It was observed that the morphology of the TaSn : PANI (1 : 0.25), (Fig. 3b) is similar to the metal oxide composite (Fig. 3b inset). However, as the amount of PANI increased, the morphology changed into porous, fiber like structure with several irregular voids. From Fig. 3c, it is clearly seen that Ta_2_O_5_–SnO_2_ particles are well dispersed over PANI matrix. When the amount of PANI was still higher (75%), the morphology was similar to PANI structure (Fig. 3d). The gas sensing studies demonstrated a high sensitivity with TaSn : PANI (1 : 0.50) composition. Therefore, all further characterizations were performed using the optimized composition.

**Fig. 4:**
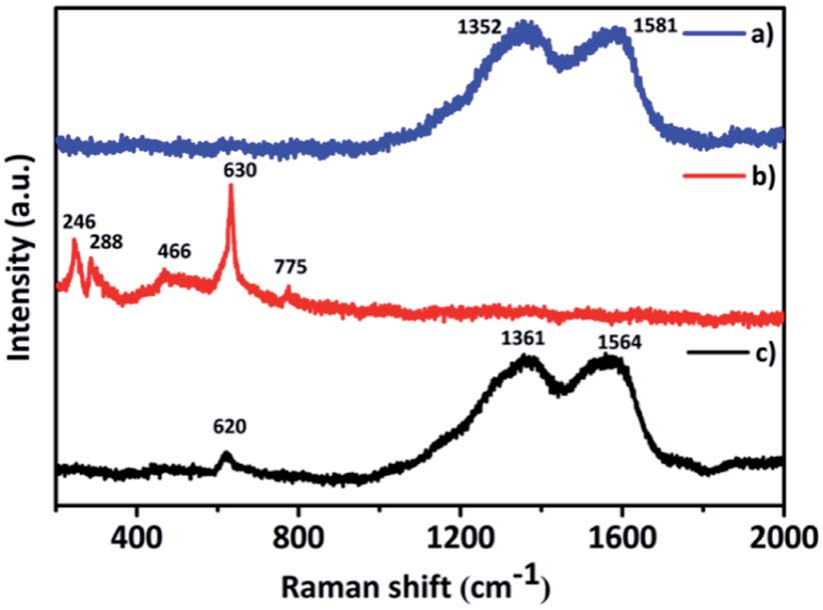
RAMAN spectra of (a) PANI, (b) (Ta_2_O_5_–SnO_2_) composite, (c) TaSn : PANI (1 : 0.50).

The interaction between the PANI and the inorganic composite Ta_2_O_5_–SnO_2_ was examined by Raman spectroscopic measurements. The two characteristic Raman vibration peaks at 1352 and 1581 cm^−1^ in PANI (Fig. 4a) could be attributed to the C–N benzenoid stretching vibration and C–C, C=C quinoid stretching vibration, respectively.^11,34^ In the Raman spectra of Ta_2_O_5_–SnO_2_ (Fig. 4b), the peaks at 466, 630 and 775 cm^−1^ are assigned to Eg, A1g and B2g modes of SnO_2_. A1g and B2g modes which are related to Sn–O bond vibrations and Eg is associated with vibrations of oxygen.^35,36^ The Raman band at 246 and 288 cm^−1^ could be due to the O–Ta–O bending vibrations of TaO_6_ octahedra.^37^ Raman spectra of the hybrid material (Fig. 4c) consist of peaks corresponding to both PANI and the Ta_2_O_5_–SnO_2_. There is a slight peak shift appeared in the hybrid material which could be due to the defects in the hybrid structure and improved oxygen vacancies.^35^ Similarly, the peaks of PANI was broadened which may be because of the interaction of PANI with the Ta_2_O_5_–SnO_2_ composite.^34^

Photoluminescence (PL) emission spectra of hybrid, PANI and (Ta_2_O_5_–SnO_2_) nanocomposite show that (Fig. S2†) the PL intensity of hybrid material is quenched compared to the (Ta_2_O_5_–SnO_2_) composite. The decrease in PL intensity is correlated to the structural defects which form non-radiative centers resulting in quenching of emission intensity.^38,39^ Therefore, the hybrid material could be comprised of defects which could help in better sensing performance. Some studies have proven that defects in terms of vacancies, dislocation of atoms, interstitial atoms contribute greatly in augmenting sensing response.^40–42^ The defect centers provide active sites for chemical activity.

### 3.2 Surface composition analysis

**Fig. 5:**
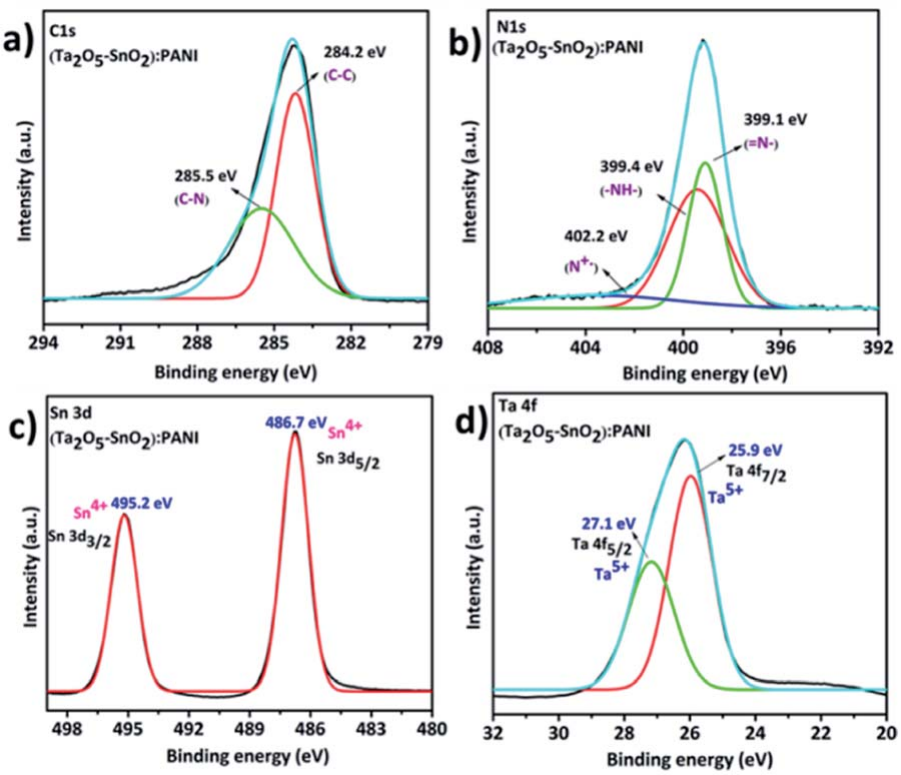
High resolution XPS spectra of (a) C 1s, (b) N 1s, (c) Sn 3d, (d) Ta 4f of hybrid TaSn : PANI.

The XPS analysis confirmed the successful formation of hybrid TaSn : PANI composite. The elemental composition of the hybrid composite was validated using XPS survey spectra (Fig. S3†) which indicated the peaks corresponding to the elements C, N, O, Sn and Ta at their respective binding energies. Fig. 5a shows the high resolution XPS C 1s spectra of the hybrid material which is resolved into two peaks. The peak at 284.2 eV corresponds to the C–C bond of PANI and the peak at 285.5 eV represents C–N bonds in PANI.^43–45^ Further, Fig. 5b shows the N 1s spectra of the hybrid composite. The N 1s spectra were deconvoluted into three peaks. The peak at 399.1 eV is assigned to benzenoid amine (=N–), 399.4 eV which represents quinoid amine (–NH–) and 402.2 eV is ascribed to nitrogen cation radical.^44,45^ In addition, the XPS spectra also revealed the presence of Sn and Ta in the hybrid material. The two peaks at 486.7 eV and 495.2 eV in Fig. 5c belong to Sn 3d5/2 and Sn 3d3/2 energy states. These energy states confirm that Sn is in the +4 oxidation state. Further, high resolution XPS spectra of Ta were resolved into two peaks (Fig. 5d) and the peaks at 25.9 eV and 27.1 eV represents Ta 4f7/2 and Ta 4f5/2 respectively. These values suggest that the Ta is in +5 oxidation state in the hybrid material.^46^

**Fig. 6:**
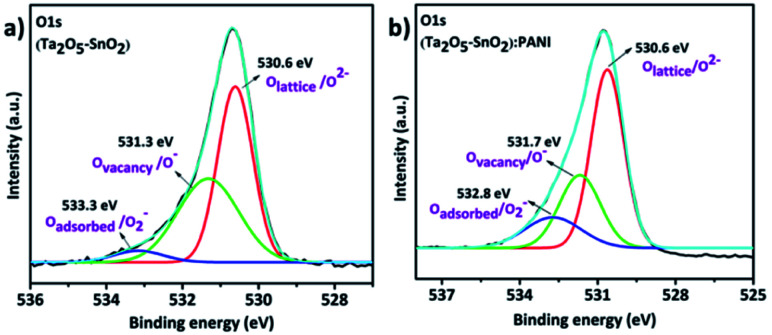
High resolution O 1s XPS spectra of (a) hybrid TaSn : PANI, (b) (Ta_2_O_5_–SnO_2_).

In addition, the oxygen vacancies were determined using O 1s spectra (Fig. 6a and b). The O 1s spectra of hybrid and the inorganic (Ta_2_O_5_–SnO_2_) composites contain different types of oxygen species. To understand the spectral features of the O 1s spectrum, multiple Gaussian peak fitting was performed. The main peak at 530.6 eV is assigned to the oxygen in the crystal lattice (O_lattice_/O^2−^). The peaks at 531.7 eV (in the hybrid composite) and 531.3 eV (in Ta_2_O_5_–SnO_2_) are related to non-stoichiometric oxygen, which is due to oxygen-deficient regions caused by oxygen vacancies (O_vacancy_/O^−^) or oxygen interstitials. Besides, the hybrid composite shows a significant amount of chemisorbed or dissociated oxygen species at 532.8 eV.^47–49^ It is noticed that the hybrid material has a high intense peak corresponding to lattice oxygen vacancies and adsorbed oxygen species. This could be due to the structural defects associated on formation of hybrid material which further help in improving the sensing performance.

### 3.3 Gas sensing studies

**Fig. 7:**
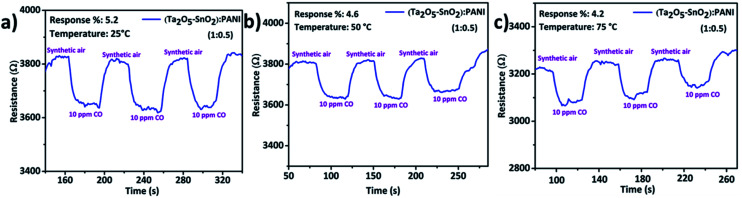
Gas sensing response and recovery curves of TaSn : PANI (1 : 0.50) nanocomposite for the detection of 10 ppm CO gas at (a) RT °C, (b) 50 °C, (c) 75 °C.

The pellets of hybrid TaSn : PANI were exposed to various concentrations of CO gas in a two probe sensing set up. Different compositions such as 1 : 0.25, 1 : 0.50 and 1 : 0.75 weight ratios of TaSn : PANI were initially tested for 10 ppm CO gas. During the dynamic sensing studies, synthetic air was used as carrier gas. Sensing response was evaluated by measuring the change in the resistance of the material before and after exposure to target gas. CO response of the hybrid composite was calculated using the following formula,

1 % response = [(*R*_air_ − *R*_gas_)/*R*_air_] × 100

where *R*_air_ is the resistance of the material before passing target gas and *R*_gas_ is the resistance on exposing to target gas. One of the main advantages of this study is that all the sensing measurements were performed at a very low operating voltage of 1 V. On exposing to CO gas, the resistance of the material decreased and interestingly the hybrid material could sense very low concentration CO gas (∼1 ppm) even at room temperature. By optimizing the experimental conditions and compositions, the 1 : 0.50 weight ratio of TaSn : PANI was found to be the most suitable for CO sensing when compared to other compositions (1 : 0.25, 1 : 0.75). All the compositions demonstrated very good sensitivity to 10 ppm CO at room temperature; however TaSn : PANI (1 : 0.50) showed high % response of 5.2% along with well-defined response and recovery curves(Fig. 7). This composition exhibited highest response to 10 ppm CO at room temperature among all other compositions. The sensing results of other compositions are given in Fig. S4 and S5† respectively. The material showed promising response up to 3 cycles with fast response and recovery time (Table 1). Remarkably, the individual inorganic composite and PANI were not sensitive to CO at room temperature; however the combination of organic–inorganic hybrid composite has demonstrated a significant improvement in the sensing performance. The dynamic range of sense for each composition is listed in Table S1.†

**Table 1:** CO sensing results of hybrid TaSn : PANI (1 : 0.50) composite

Operating temp. (°C)	% response	Response time (s)	Recovery time (s)
RT	5.2	14	13
50	4.6	18	15
75	4.2	15	14
100	2.9	13	16
125	1.5	14	15
150	No response	—	—

**Fig. 8:**
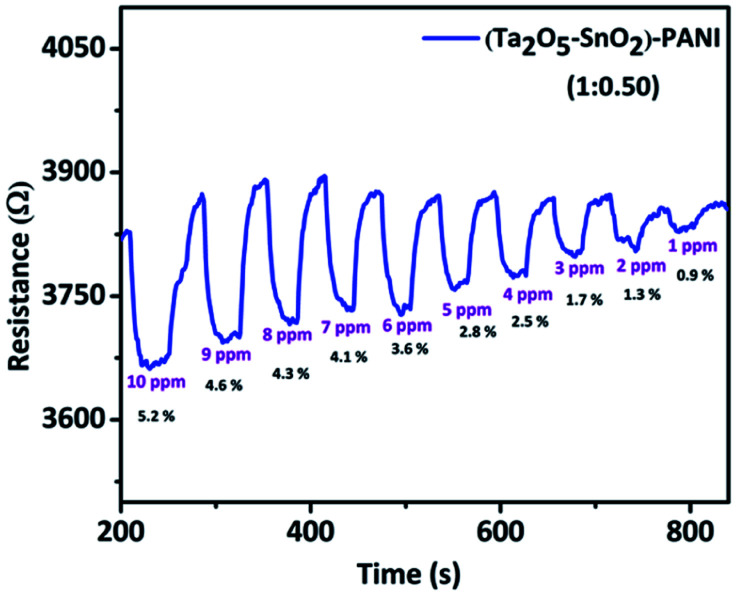
Sensitivity of TaSn : PANI (1 : 0.50) hybrid material at different concentrations of CO.

Further, we tested the gas response at different concentrations of CO gas to observe the lowest concentration of the gas that the hybrid material could detect. It is noticed that a minimum detection limit of 1 ppm CO was achieved with the hybrid material (Fig. 8). It was observed that the sensor shows linear response within the tested concentration range (1–10 ppm) (Fig. S6†).

**Fig. 9:**
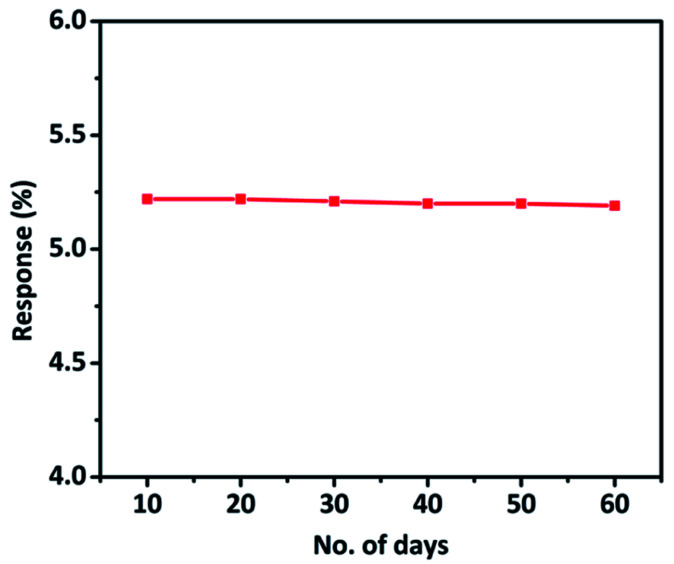
CO sensing response of TaSn : PANI (1 : 0.50) hybrid material up to 60 days (with the interval of 10 days).

The stability study of the hybrid material was performed every 10 days up to 60 days and the CO sensing response of the hybrid material was monitored. The material demonstrated exceptional stability and retained its sensing properties even 60 days after the synthesis (Fig. 9). We studied the stability of 3 sensors as a function of time. All the sensors exhibited long term stability without much loss of sensitivity to CO gas. A minimum error of ±1.1% was observed during the sensing measurements (Fig. S7†).

**Fig. 10:**
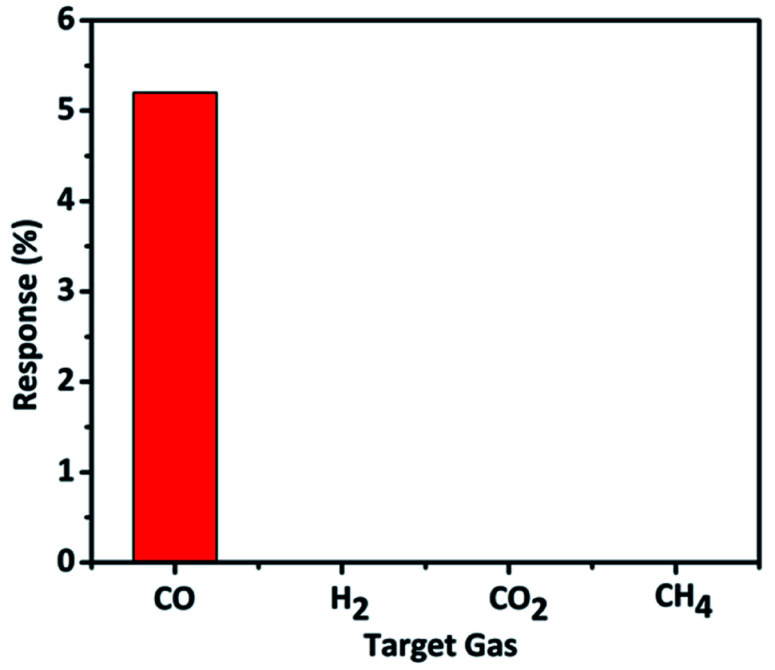
Selectivity study of TaSn : PANI hybrid material.

The selectivity study revealed that the material is particularly sensitive to CO gas and showed high selectivity only to CO and not for the other interfering gases such as H_2_, CH_4_ and CO_2_ gases (Fig. 10). We studied selectivity with common interfering gases along with CO such as H_2_, CO_2_, and CH_4_. When CO releases from automobiles or industries there are chances of presence of other gases (H_2_, CO_2_, and CH_4_) and cross-sensitivity of the sensor must be avoided in such cases. The designed sensor should be able to sense only the desired target gas at particular operating conditions. Overall, the material displayed remarkable CO gas sensing performance at mild conditions which is very crucial in practical applications. The results presented here are comparable to the best reports available in the literature for CO gas sensing.^6,29,30,50^

**Fig. 11:**
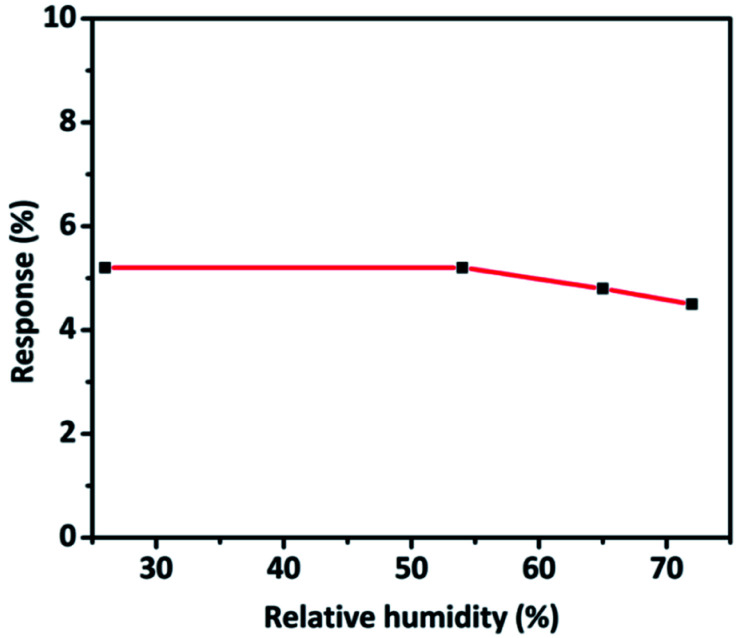
Variation of response of TaSn : PANI (1 : 0.50) sensor to CO gas at RT with relative humidity.

The effect of relative humidity on CO sensing properties of TaSn : PANI (1 : 0.50) was explored at different humidity levels. Interestingly, there was no significant decrease in the sensitivity of the material even at a higher humidity level. An insignificant change in response from 5.2% to 4.8% was observed which could be due to the adsorption of water molecules that obstruct the adsorption of target gas molecules on the surface of the sensor (Fig. 11).^6,29^

### 3.4 Gas sensing mechanism

The gas sensing mechanism of semiconducting metal oxides is well explored in the literature which is mainly based on the electronic interaction of sensor material with the adsorbed gas molecules.^10,51,52^ The oxygen molecules adsorbed on the surface of the sensing material capture electrons from the conduction band and convert into oxygen anion species (O^2−^, O^−^, O_2_^2−^) (eqn (2)–(4)).^11^ This results in a decrease in electron concentration in the sensor material. However, on exposing to the target gas, (CO gas in the present study), the CO gas molecules will react with oxygen anion species to give CO_2_ molecules and return electrons to the metal oxide (eqn (5) and (6)) which tends to increase the conductivity of the sensor material by providing more number of electrons to the material.

2 O_2(ads)_ + e^−^ ↔ O_2−_

3 O_2−_ + e^−^ ↔ 2O^−^

4 O^−^ + e^−^ ↔ O^2−^

5 2CO + O^2−^ ↔ CO_2_ + e^−^

6 CO + O^−^ ↔ CO_2_ + e^−^

This is a common phenomenon that takes place in chemiresistive based semiconducting metal oxides. However, the improved gas sensing performance of the TaSn : PANI hybrid material to sense low concentration CO at room temperature could be due to several factors such as high conducting properties of PANI, increased oxygen vacancies, and most importantly due to the formation of hetero-junction resulting in the synergistic interaction between the PANI and the inorganic (Ta_2_O_5_–SnO_2_) composite. Herein, we propose a possible mechanism which could be responsible for the superior sensing performance of the hybrid material. The (Ta_2_O_5_–SnO_2_) inorganic nanocomposite is a n-type semiconducting oxide and PANI is a p-type material. Therefore, a strong n–p–n hetero-junction would be present between the inorganic composite and the polyaniline molecules.^24,53^ Due to the hetero-junction effect, the movement of the electrons and holes takes place across the interface of two materials to attain equilibrium. This continuous movement of charge carriers may lead to a charge transfer complex and the inherent conducting properties of PANI further enhance the sensing performance of the hybrid material. It is evident from the literature that the formation hetero-junction reduces the activation energy and enthalpy of physisorption with excellent electron donating characteristics.^26,53^ In addition, the combination of organic–inorganic composites creates defects in the structure and increases the number of active sites in the material. Overall, these synergistic effects of TaSn : PANI hybrid material are responsible for the improved sensitivity of the hybrid nanocomposite.

### 3.5 Comparison of CO sensing performance of hybrid TaSn : PANI composite with other materials reported recently for CO sensing (based on chemiresistive principle)

The CO sensing properties of hybrid TaSn : PANI was compared with the some of the best chemiresistive sensor materials reported in literature recently (Table 2). Except for a few reports, many of the articles report the CO detection above threshold limit value (TLV) and they operate at high operating temperatures which severely hamper their commercialization. In the present work, we demonstrated a remarkable sensing of TaSn : PANI for detecting low concentration CO (up to 1 ppm) at room temperature with good sensitivity, selectivity. A stable, reproducible response was obtained with fast response and recovery time. Herein, we could achieve the detection of very low concentration CO at room temperature and the results obtained are at par with the best reports available for CO sensing in the literature.

**Table 2:** Comparison of sensing performance with literature reports

Material	Temp. (°C)	Concentration (ppm)	Ref.
SnO_2_–Au–WS_2_	RT	50	6
PANI–MWCNT	RT	500–1000	30
PANI–Co_3_O_4_	RT	75	29
Alpha-Fe_2_O_3_–rGO	RT	10	50
Alpha-Fe_2_O_3_–rGO QD	RT/60	105 ppb	54
rGO	RT	10	55
Au–SnO_2_–CNT	RT	500–2500	56
Co_3_O_4_	100	5	7
MOF derived Co_3_O_4_	150	500 ppb	57
Au–SnO_2_/In_2_O_3_	200	10	58
SnO_2_–CuO	180	5–500	59
Pd/SnO_2_	100	100	60
Pt/WO_3_	125	100–500	61
Au–SnO_2_/ZnO	25–200	50	62
Ga/ZnO	250	50	63
Alpha-Fe_2_O_3_	250–400	10–100	64
Ta_2_O_5_–SnO_2_	50	5–10	65
**Ta_2_O_5_–SnO_2_–PANI**	**RT**	**1–10**	**Present work**

## 4. Conclusions

An inorganic–organic hybrid nanocomposite of TaSn : PANI was synthesized by oxidative polymerization complex method and explored for very low concentration CO detection at room temperature. The p-XRD analysis revealed that the inorganic metal oxide composite (Ta_2_O_5_–SnO_2_) retained its crystal structure even after incorporating with PANI. Furthermore, FTIR and RAMAN characterizations confirmed the presence of PANI in the synthesized hybrid composite. FESEM analysis demonstrated the porous fibrous network like surface morphology of the PANI and different compositions of hybrid material. XPS analysis confirmed the successful combination of PANI and (Ta_2_O_5_–SnO_2_) nanocomposite oxide. In addition, the high resolution XPS spectra revealed the details about oxidation states of Sn, Ta, C and N bonds present in the PANI. From the O 1s spectra of XPS analysis, we observed a higher number of oxygen vacancies in case of hybrid composite. Gas sensing studies of the TaSn : PANI hybrid showed significant improvement in sensing a low concentration (up to 1 ppm) CO gas at room temperature. The amount of each component was optimized and 1 : 0.50 weight ratio of TaSn : PANI hybrid material exhibited high % response of 5.2% than the other compositions. A stable, reproducible response and recovery curves were obtained with very good sensitivity, high selectivity and fast response and recovery time. Thus, using a combination of both inorganic–organic materials results in an efficient CO sensor. A possible mechanism was explained based the conducting properties of PANI, improved oxygen vacancies and synergistic interaction between the p–n hetero-junction formed between PANI and (Ta_2_O_5_–SnO_2_).

## Conflicts of interest

There are no conflicts of interest to declare.

## Supplementary Material

RA-012-D2RA00602B-s001
