# Supplementary material for: Polyaniline/(Ta2O5–SnO2) hybrid nanocomposite for efficient room temperature CO gas sensing
Source: RSC Adv. 2022 May 25;12(25):15759–66. doi: 10.1039/d2ra00602b (PMC9132034; doi:10.1039/d2ra00602b)
Supplement: RA-012-D2RA00602B-s001 [file RA-012-D2RA00602B-s001.pdf]

Electronic Supplementary Material (ESI) for RSC Advances.
This journal is © The Royal Society of Chemistry 2022

Electronic Supporting Information

Polyaniline/(Ta_2O_5-SnO_2) Hybrid Nanocomposite for Efficient Room Temperature CO Gas Sensing

Chethana Aranthady,[a,b] Ganapati V. Shanbhag,[a] Nalini G Sundaram [a,c]*

[a] *Materials Science and Catalysis Division, Poornaprajna Institute of Scientific Research, Devanahalli-562164, Bengaluru, India*
[b] *Graduate Studies, Manipal Academy of Higher Education, Manipal - 576104, Karnataka, India.*

[c] *Department of Chemistry, St. Josephs's Colleges (Autonomous), Bengaluru-560027, India*

**Corresponding author: nalini@poornaprajna.org*

Table S1: Dynamic range of sense (maximum and minimum CO gas response) for each composition at different operating temperatures.

Composition	Operating temp. (°C)	% response	Response time (s)	Recovery time (s)
TaSn:PANI(1:0.25)	**R**RTT	4.7	15	13
	50	4.2	15	12
	75	4.1	16	14
	100	2.3	13	15
	125	0.09	16	18
	150	No response	-	-
TaSn:PANI(1:0.50)	**R**RTT	5.2	14	13
	50	4.6	18	15
	75	4.2	15	14
	100	2.9	13	16
	125	1.5	14	15
	150	No response	-	-
TaSn:PANI(1:0.75)	**R**RTT	3.8	22	20
	50	3.5	23	22
	75	2.3	20	24
	100	1.8	22	21
	125	No response	-	-
	150	No response	-	-

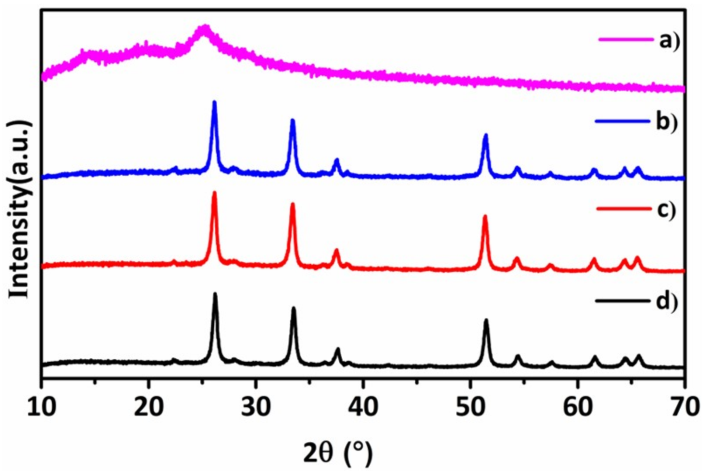

Figure S1: p-XRD patterns of a) PANI, b) TaSn:PANI (1:0.75), c) TaSn:PANI (1:0.25), d) (Ta_2O_5-SnO_2).

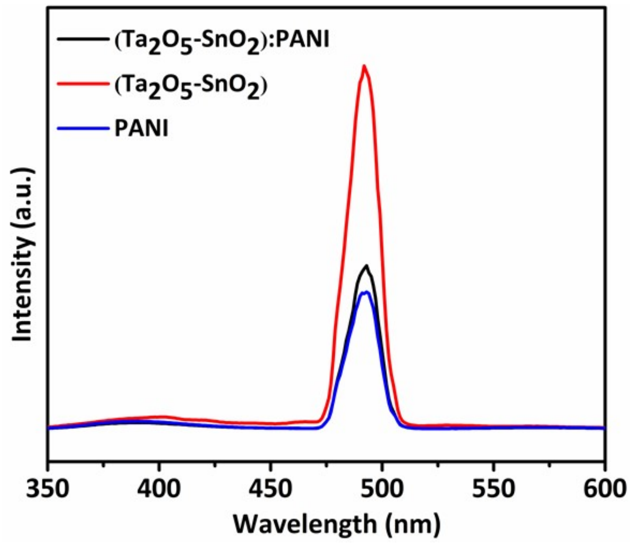

Figure S2: PL emission spectra of hybrid TaSn:PANI (1:0.50), PANI and (Ta_2O_5-SnO_2).

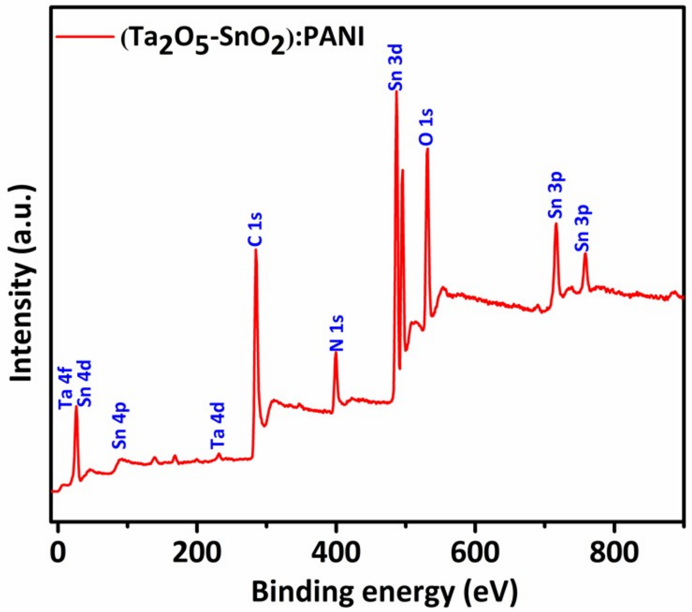

Figure S3: XPS survey spectra of hybrid TaSn:PANI (1:0.50) composite.

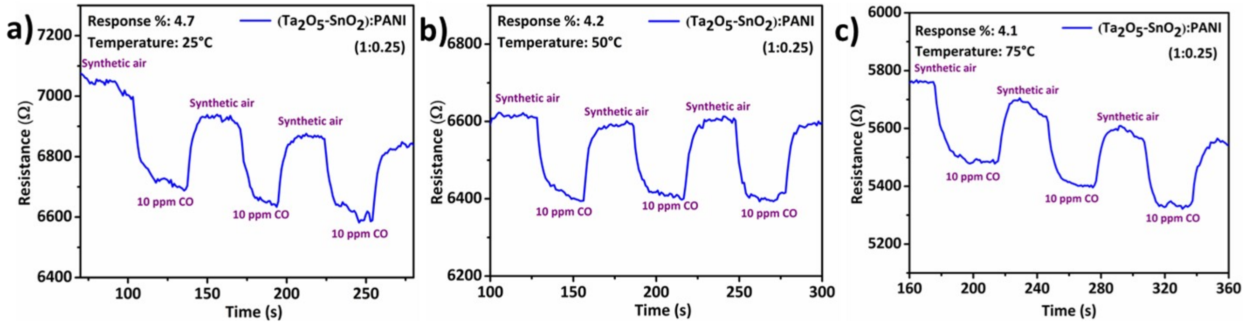

Figure S4: Gas sensing results of TaSn:PANI (1:0.25) nano composite for the detection of 10 ppm CO gas at a) RT °C, b) 50 °C, c) 75 °C.

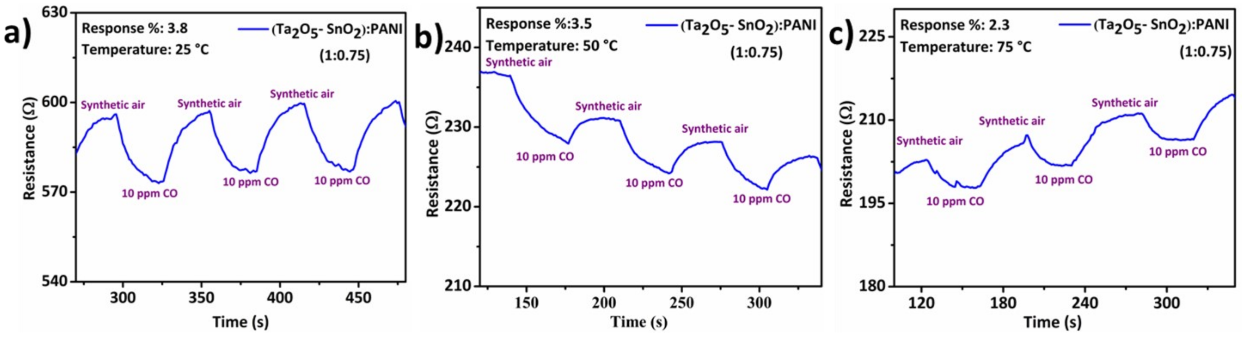

Figure S5: Gas sensing results of TaSn:PANI (1:0.75) hybrid nanocomposite for the detection of 10 ppm CO gas at a) RT °C, b) 50 °C, c) 75 °C.

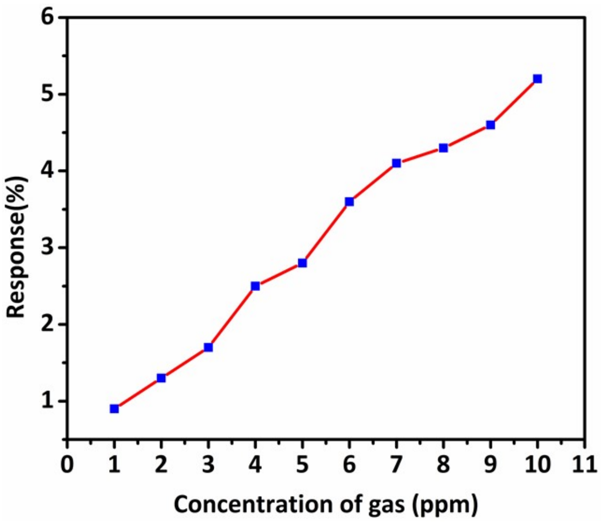

Figure S6: Sensor response with reference to concentration of CO.

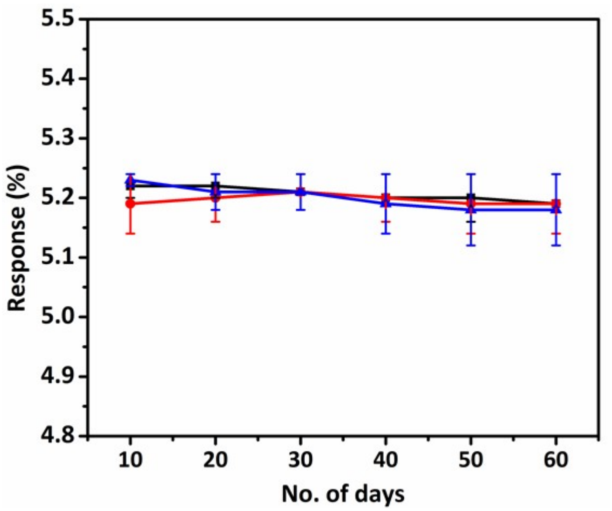

Figure S7: Sensor stability studies for 3 sensors as a function of time with error calculation.
